# Remembered or Forgotten?—An EEG-Based Computational Prediction Approach

**DOI:** 10.1371/journal.pone.0167497

**Published:** 2016-12-14

**Authors:** Xuyun Sun, Cunle Qian, Zhongqin Chen, Zhaohui Wu, Benyan Luo, Gang Pan

**Affiliations:** 1 College of Computer Science and Technology, Zhejiang University, Hangzhou, Zhejiang, China; 2 The First Affiliated Hospital of Medical School, Zhejiang University, Hangzhou, Zhejiang, China; University of Zurich, SWITZERLAND

## Abstract

Prediction of memory performance (remembered or forgotten) has various potential applications not only for knowledge learning but also for disease diagnosis. Recently, subsequent memory effects (SMEs)—the statistical differences in electroencephalography (EEG) signals before or during learning between subsequently remembered and forgotten events—have been found. This finding indicates that EEG signals convey the information relevant to memory performance. In this paper, based on SMEs we propose a computational approach to predict memory performance of an event from EEG signals. We devise a convolutional neural network for EEG, called ConvEEGNN, to predict subsequently remembered and forgotten events from EEG recorded during memory process. With the ConvEEGNN, prediction of memory performance can be achieved by integrating two main stages: feature extraction and classification. To verify the proposed approach, we employ an auditory memory task to collect EEG signals from scalp electrodes. For ConvEEGNN, the average prediction accuracy was 72.07% by using EEG data from pre-stimulus and during-stimulus periods, outperforming other approaches. It was observed that signals from pre-stimulus period and those from during-stimulus period had comparable contributions to memory performance. Furthermore, the connection weights of ConvEEGNN network can reveal prominent channels, which are consistent with the distribution of SME studied previously.

## Introduction

The brain is one of the largest and most complex organs in human body. In order to decode specific cognitive states from brain activity, many efforts have been made, for instance, detecting concealed true thoughts when answering questions [1], decoding features of motor behavior [2], distinguishing specific perceived stimulus from several candidate stimuli [3, 4], inferring visual imagery in dreams [5] and identifying traces of individual episodic memories [6].

Memory formation is an important cognition process. It enables us to store information, accumulate experiences and learn from experiences to guide our behaviors. Understanding cognitive states related to memory formation is essential to investigate underlying brain mechanisms and even improve our memory performance. As a result, decoding neural activities during memory process has aroused much interest in the cognitive neuroscience community. Neural activities relevant to memory formation can be observed using different physical measurements, e.g. fMRI (functional magnetic resonance imaging) for BOLD activity and EEG (electroencephalography) for electrophysiological activity. These measurements help us analyze the process of memory. Among different measurements, EEG is widely used in disease diagnosis [7], neuroscience and psychological research [8, 9] for its practical advantages, such as noninvasion, mobility and relatively inexpensive devices. Specifically, EEG can be used to reveal the correlation between memory cognition process and subsequent memory performance. Many studies have found statistical differences in EEG before or during learning between subsequently remembered and forgotten events, which are defined as subsequent memory effects [10–15]. These differences have shown that brain signals relevant to an event can contribute to successful memory encoding and later recollection.

In this paper, we addressed prediction of subsequent memory performance using EEG recorded during memory process. While the findings mentioned above used multi-instance EEG signals to reveal the SME, we try to make single-instance analysis of SME for predicting memory performance. By predicting whether an event will be remembered or forgotten later, effective actions could be taken to help us remember new knowledge and improve the efficiency of learning. It could also help people with memory disorder and even cognitive impairment with new prevention, diagnosis and rehabilitation methods.

From the computational perspective, prediction of subsequent memory performance is a typical binary pattern recognition problem with the two classes of subsequently remembered and forgotten events. For a general pattern recognition problem, meaningful features need to be extracted to maximize the differences between different classes and then a classifier uses hand-crafted features to predict which class they belong to. However, for most problems, it’s difficult to design features exactly useful for classification. And feature extraction and classification are two separate phases, which make it complex for realization and optimization.

This paper proposed a convolution neural network for EEG, named ConvEEGNN, to predict whether an event will be remembered or forgotten later. It can combine feature extraction and classification as a whole. We conducted an auditory memory task, consisting of a study phase and a memory test, to verify ConvEEGNN. EEG signals before and during an auditory event were recorded in the study phase. According to information before and during an event, the average prediction accuracy of 72.07% was achieved.

## Related Work

Many studies have been carried out to investigate the correlation between memory performance and episodic memory process, which is a form of long-term memory. It has been recently shown that item-related, state-related and task-related neural activity all can affect whether an event will be remembered or forgotten later [11, 14–16], updating the theoretical explanation of memory encoding. Meanwhile, techniques and methods from pattern recognition have been embraced to help analyze and interpret memory process, such as multi-voxel pattern analysis (MVPA), common spatial pattern (CSP), support vector machine (SVM) and linear discriminant analysis (LDA).

With the improvement of neural measurements, increasing interest has been aroused in researches on the neural systems responsible for episodic memory encoding. Episodic memory is the memory of events in our own personal past. It is defined as the conscious knowledge of temporally dated, spatially located, and personally experienced events or episodes [17]. The components of an event such as words or pictures requiring discriminative responses are items or stimuli [18]. Item-related activity, state-related activity and task-related activity influence episodic memory encoding. Sanquist et al. found that item-related activity affects the efficacy of episodic memory encoding of a stimulus by means of segregating item-related neural responses with different later memory performances and identifying the features of the responses correlated with successful encoding of visual stimuli [11]. In addition to item-related activity, Otten et al. showed that state-related activity, that is, neural activity sustained across a succession of stimulus events, influences memory encoding by finding the relation between the mean level of activity across a task block and the number of visually presented words subsequently remembered from that block [16]. After that, the investigation by Otten et al. on task-related neural activity preceding a stimulus event suggests that task-related activity is also predictive of successful encoding for visual and auditory events [14, 15].

Task-related activity and item-related activity constitute SMEs and both showed statistical differences in EEG response to a stimulus between the subsequently remembered and forgotten items for respective pre- and during-stimulus period. An fMRI study also presented SMEs in the level of hippocampal BOLD activity before item presentation [18]. Therefore, EEG and fMRI signals have shown correlation with subsequent memory performance in group analysis.

Currently, little study has been carried out to predict subsequent memory performance for single stimulus in every participant. Methods from pattern recognition have been used for this prediction problem [19–21]. In a recent fMRI study, MVPA has been used to predict subsequent memory performance for 19 participants according to the period of encoding phonogram stimuli [19]. The analysis consisted of 3 stages. First, MVPA-based voxel-wise search for the clusters in the medial temporal lobe was conducted to find the signals contained the most information about subsequent memory performance. Then, a classifier function in MVPA was trained using the extracted pattern vectors from the selected clusters. Finally, the trained classifier predicted subsequent memory performance with approximately 66% accuracy. However, the slowness of the vascular response may influence the precise selection for the encoding period and lead to impure signals for analysis. Using EEG, a more mobile and affordable noninvasive method for monitoring brain activity, Noh et al has identified subsequent episodic memory performance on single-trial neocortical dynamic activity recorded before and during item presentation from 18 participants [20]. CSPs were used to learn the spectral features of pre- and during-stimulus SME, which were classified respectively by two soft margin support vector machines (v-SVM). Another classifier using LDA was trained to learn the temporal features of during-stimulus SME. By combining the results from the three separate classifiers and also combining information from the pre- and during-stimulus periods, the overall prediction accuracy achieved 59.6%. The accuracy using EEG signals might be relative lower than that using fMRI signals because of the higher spatial resolution of fMRI. In a recent EEG study with Sternberg Working Memory Task (SWMT), SVM has been used to identify signal features associated with working memory performance for 40 schizophrenia adults and 12 healthy adults [21]. Using continuous wavelet transform (CWT), EEG of each trial was analyzed to extract time-frequency and spatial features, including 5 frequency bands at 4 processing stages and 3 scalp sites. Then, 1-norm SVM was used as a classification approach to predict working memory performance according to the extracted 60 features. This approach predicted SWMT trial performance with 84% accuracy in healthy adults and 74% accuracy in schizophrenia adults. Overall, the related work mentioned in [19–21] all used methods from pattern recognition to extract information from data and predict memory performance for each stimulus.

## Materials

To evaluate the proposed prediction approach, we adopted an auditory memory task [15], during which EEG responses to auditory stimuli were recorded for predicting memory performance at a later time. Participants were paid to take part in the auditory memory task. The experimental procedure consists of a study phase and a memory test. In the study phase, participants listened to a word after a cue and made semantic (animate or not) judgments about the word. In the memory test, words in the study phase had to be discriminated from new words. Participants were asked to make a judgment from five candidates (1.definitely familiar, 2.possibly familiar, 3.uncertain, 4.possibly unfamiliar, and 5.definitely unfamiliar), and press a key from 1 to 5 accordingly.

This experiment was approved by the Ethical Committee of the First Affiliated Hospital, Zhejiang University School of Medicine. All of the healthy participants obtained written informed consent before the experiment. The experiment was performed in accordance with the guidelines issued by the Ethical Committee of the First Affiliated Hospital, Zhejiang University School of Medicine.

### Participants

Twenty-two right-handed healthy participants (16 females and 6 males, 21-32 years old) were enrolled, who are native Chinese speakers without neurological or psychiatric history. Out of the 22 participants, 13 were excluded based on the two criteria below:

1. participants who remembered or forgot less than 15 words were excluded to ensure the number of samples for algorithm training [15];
2. participants with high response bias (response bias>0.2) were excluded for the high possibility of recognizing a new word as a studied one. Response bias, which was proposed in [22], is a criterion to exclude the participants with high possibility of choosing “definitely familiar” or “possibly familiar” when facing “uncertain” words in a memory test.

In our study, we excluded 6 participants for their high response bias and 7 participants who forgot less than 15 words. As a result, 9 participants were for our evaluation (4 females and 5 males).

### Stimuli

Study and test list were drawn from a pool of 200 concrete nouns with a length of two Chinese characters and a frequency of 0-500 occurrences per million from [23]. Each word was recorded in spoken form (male voice, 44.1 kHz, mean duration 650 millisecond (msec), range 600-700 msec). A study list consisted of 100 words with random order. A test list contained 200 words, made up of a random sequence of 100 studied and 100 new words. Auditory cue is a 44.1 kHz pure tone (200 msec duration).

### Task and Procedure

The experiment involved a study phase, followed by a memory test. Participants were first prepared for the recording of brain activity, namely EEG. EEG was recorded with a 32-channel BrainCap MR (www.brainproducts.com/filedownload.php?path=downloads/Electrode_Caps/BrainCapMR32_Names.jpg) using a 32-channel Brain-Amp Amplifier (Brain Products, Munich, Germany, 5 kHz sampling). FCz was used as the online reference, and Iz (an electrode placed just anterior to Oz) served as ground. Vertical eye movements were recorded from VEOG (vertical electrooculogram) placed at the supra- and infraorbital ridges of the right eye, and horizontal eye movements were recorded from HEOG (horizontal electrooculogram) placed at the outer canthus of each eye. Signals were amplified and band-pass filtered between 0.01 and 70 Hz (Contact Precision amplifier; 3 dB roll-off) with a notch filter at 50 Hz, and digitized at 500 Hz. Impedances of recorded electrodes were kept below 5k Ohm. In order to suppress the influence to EEG recording brought by muscular movements, the participants were instructed to reduce their facial and head movements during signal recording. In addition, any facial or head movement was inspected and marked during the experiment.

During the study phase, the participants were instructed to create a mental image denoted by each word heard via headphones and make a semantic judgment about the word. An auditory cue presented 1.5 seconds (sec) before each word, indicating the upcoming of a stimulus. Judgments were saved by depressing a corresponding button with the index finger of left or right hand. A practice block helped the participants get used to the task at the beginning of the study phase. The study list was presented across four blocks. Each block consisted of 25 words and was separated by a break of five seconds.

**Fig 1:**
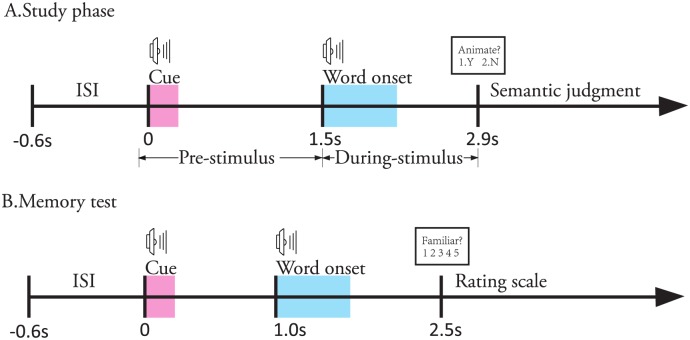
Timings of the auditory memory task in the study phase (A) and memory test (B). The two shaded areas in the study phase are the lasting time for an auditory cue and an auditory word respectively. The participants were instructed to make a semantic judgment about the word with the “animate or not” question showing on a screen. In the memory test, the two shade areas have the same meaning as those in the study phase. The participants made a judgment about the scale of familiarity by pressing a key from 1 to 5. The ConvEEGNN approach is designed to predict whether the participant remembered the word in the study phase by analyzing the EEG recorded from the study phase.

The participants were given a memory test approximately 45 minutes after the end of the study phase. In the memory test, an auditory cue presented 1 sec before word onset, and then all the words in the study list were re-presented as well as new words not encountered previously. The participants were required to decide whether they had experienced the word in the study phase and to indicate the confidence in their decision by pressing a number key from 1 to 5 for a rating scale (1.definitely familiar, 2.possibly familiar, 3.uncertain, 4.possibly unfamiliar, and 5.definitely unfamiliar). Fig 1 shows timings of the auditory memory task in the study phase and the memory test with a fixed inter-stimulus interval (ISI) for 0.6 sec.

### EEG Pre-processing

The recorded EEG data from the study phase of the experiment were pre-processed by the following six steps:

1. re-reference: EEG were algebraically re-referenced to linked mastoids;
2. filtering: the data were band-pass filtered between 0.05 and 15 Hz (48 dB roll-off, zero phase shift IIR filter) to remove low-frequency noise [15];
3. blink detection and correction: in order to remove eye movement artifacts, a standard regression technique [24] was used to estimate and correct the contribution of artifacts to the waveforms;
4. segmentation: data from -0.1-2.9s duration around events of interest were further segmented into several trials. The start point of a segment is 100msec before cue onset (0s), namely -0.1s. The end point of a segment is 2.9s, right before making a judgment about a word;
5. baseline correction: each segment was referred to a 100-msec period before cue onset.
6. artifacts rejection: trials containing EEG drifts (±50 *μ*V) [15], marked facial movements and head movements were excluded from further analysis.

After the forth step, EEG data were segmented to ERP (event-related potential). ERP is the measured brain response that is the direct result of a specific sensory, cognitive, or motor event [25]. It is an EEG response to a stimulus. ERPs provide a continuous measure of processing between a stimulus and an EEG response, making it possible to determine which period is being affected by a specific stimulus [25]. The data after pre-processing are available publicly (https://github.com/panlab/ConvEEG).

### SME of Our Auditory Memory Task

**Fig 2:**
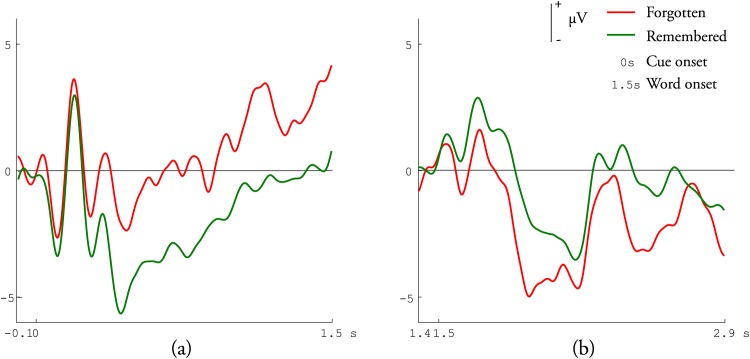
Grand-averaged ERP waveforms for remembered/forgotten words at a representative frontal electrode site (site Fp1 of the 10/10 system). Positive values are plotted upwards. (a) Pre-stimulus neural activity of auditory events. After a cue about an upcoming word, ERPs were elicited and analyzed by overlaid according to whether the word was remembered or forgotten. (b) During-stimulus neural activity of auditory events. After an auditory presented word, ERPs were elicited and analyzed by overlaid according to the judgments made in the memory test.

To verify SME in our data, ERP waveforms after artifacts rejection for each participant were averaged into individual-averaged ERP according to whether the word was remembered or forgotten in the subsequent memory test. Trials were labeled as remembered for the words in the study list given definitely familiar or possibly familiar judgments in the memory test. And trials were labeled as forgotten for the words in the study list given uncertain, possibly unfamiliar or definitely unfamiliar judgments in the memory test. Then, individual-averaged ERPs of all the participants were further averaged into grand-averaged ERP. Finally, ERP waveforms were qualified by measuring mean amplitudes of grand-averaged ERP (Fig 2). Fig 2 shows a significant subsequent memory effect for both pre-stimulus period (t-score < 0.01) and during-stimulus period (t-score < 0.01). For pre-stimulus period, more negative-going ERPs are elicited for subsequently remembered words than forgotten ones while for during-stimulus period, more positive-going ERPs are elicited for subsequently remembered words than forgotten ones, which are in accordance with [14, 15].

## Methods

### Problem Definition

SMEs have shown that there exist differences in EEG data between the subsequently remembered and forgotten events, which may be used to predict subsequent memory performance. Here we attempt to predict remembered or not from the recorded EEG signals. We formalize it as a pattern recognition problem of two-category classification. Suppose that we have a set of n samples with their labels {(I_i_, Z_i_), i = 1, 2, …, n}, where I_i_ is a piece of EEG signals during memory process for an event (in our experiment, each word presenting is an event), and Z_i_ is the label of the sample I_i_ indicating remembered or forgotten. We want to use these samples to learn a model H: I → Z to establish the connection between the neural activities and memory performances. Therefore, for any EEG input I_0_ of an event, its memory performance $\tilde{Z}$ will be predicted by H,

$$\begin{array}{c} \tilde{Z}=H\left(I_{0}\right) \end{array}$$ (1)

A sample I_i_ usually consists of EEG data from N channels of EEG electrodes, with the temporal sampling length T of each channel.

### ConvEEGNN: Convolutional EEG Neural Network for Prediction

To predict whether an event will be remembered or forgotten, we design a convolutional neural network (CNN) for EEG, called ConvEEGNN. In general, CNN is a variant of multilayer perceptron with local connectivity and shared weights, which were inspired by biological processes [26, 27]. It can be efficient to extract underlying features and tolerate variations over space and time. It has been widely used in various applications, for example, handwriting character recognition [28], object categorization [29–32], multimedia retrieval [33], face recognition [34], and speech recognition [35, 36].

**Fig 3:**
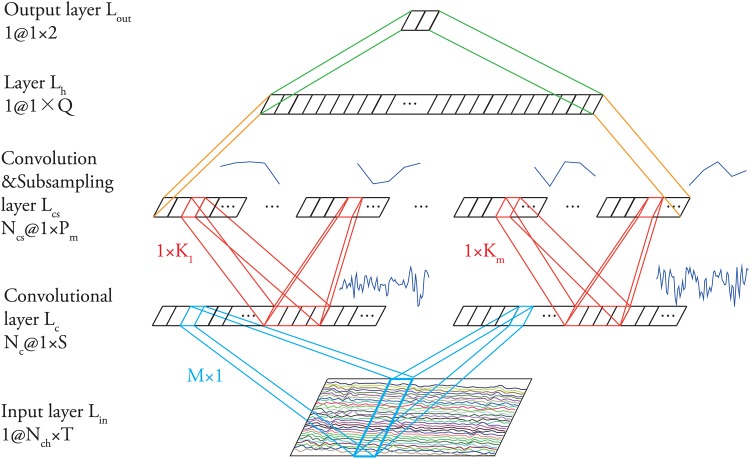
Architecture of ConvEEGNN.

Our proposed ConvEEGNN is a CNN specified for EEG understanding. Network topology of ConvEEGNN is a key feature, which may eventually affect its prediction performance. A reasonable topology can translate successive signal processing or feature extracting steps. Consequently, we design the topology for ConvEEGNN depicted in Fig 3. It contains five layers: an input layer L_in_, a spatial convolutional layer L_c_, a temporal convolutional and subsampling layer L_cs_, and two fully connected layers L_h_, L_out_. Neurons in a layer are organized in planes and the output of neurons in a plane is called a feature map. Each layer comprises one or several feature maps. Generally, for the convolution transform in Layer L_c_ and L_cs_, each neuron of a map is connected locally from the previous layer and shares the same set of weights. Layer L_cs_, L_h_ and L_out_ can be regarded as a multilayer perceptron. The architecture of ConvEEGNN is described in more detail as follows.

The input of ConvEEGNN is a matrix I, consisting of N_ch_ channels. Each channel is a time series of voltage measures with the length of T, namely, d^th^ channel is **a_d_** = [x_1_, x_2_, …, x_T_]. Therefore, the input I can be denoted as

$$\begin{array}{c} \text{I}=\left[\begin{array}{c} {\text{a}}_{1}\\ {\text{a}}_{2}\\ \vdots \\ {\text{a}}_{{\text{N}}_{\text{ch}}} \end{array}\right] \end{array}$$ (2)

The size of I is N_ch_ × T, where T corresponds to the temporal sampling length. T depends on sampling frequency and time interval for analysis.

- Layer L_in_: the input layer receives input EEG data I_i_. The input data are real values for N_ch_ channels and temporal sampling length T.
- Layer L_c_: the first hidden layer is a convolutional layer, which convolves data in the spatial domain. Neurons in the convolutional layer are organized in N_c_ feature maps, each of which has S neurons. A neuron in a feature map has M inputs connected to a M by 1 area in the input, which is the receptive field of the neuron. Accordingly, each neuron has M trainable weights and a trainable bias. To detect the same feature at all possible location on the input, all the neurons in a feature map share the same set of weights, which is called the kernel of the map, and the same bias. Therefore, L_c_ contains N_c_ × (M + 1) trainable parameters and S × N_c_ × (M + 1) connections. In this study, M is set to be N_ch_ and S is set to be T.
- Layer L_cs_: the second hidden layer is a convolutional and subsampling layer, which subsamples and transforms the data in the temporal domain. Neurons in this layer are organized in N_cs_ feature maps. The map m of L_cs_ has P_m_ neurons (m = 1, 2, … N_cs_). Each neuron in a feature map m is connected to 1 × K_m_ neighborhood in the corresponding feature map in L_c_. The 1 × K_m_ receptive fields are non-overlapping in order to down-sample the input from L_c_. L_cs_ contains $\sum_{m=1}^{m\leq N_{cs}}\left(K_{m}+1\right)$ trainable parameters and $\sum_{m=1}^{m\leq N_{cs}}P_{m}\times \left(K_{m}+1\right)$ connections. In this study, the number of maps in L_cs_ is set to be the same as that in L_c_, that is N_cs_ = N_c_.
- Layer L_h_: the third hidden layer is composed of one map of Q neurons and is fully connected to L_cs_. Each neuron has $\sum_{m=1}^{m\leq N_{cs}}\left(P_{m}+1\right)$ input parameters and connections of the same size. Q is set to be 10 in this study.
- Layer L_out_: the output layer has one map of two neurons fully connected to L_h_. The two neurons, Z_0_ and Z_1_, represent the two classes of remembered and forgotten events. This layer has 2 × (Q + 1) parameters.

In ConvEEGNN, layer L_c_ and L_cs_ play the important role in prediction. Neurons in the spatial convolutional layer L_c_ are organized in maps and each neuron has M inputs connected to a M × 1 area in the input layer, that is, the receptive field of the neuron. The weight vector connecting the receptive field and each neuron in layer L_c_ is the kernel for this layer. The stride of the kernels for this layer is set to one. All the neurons in the same map share the same set of weights. Thus, all the neurons in one map of L_c_ perform the spatial filtering on different channels of the data and result in a channel combination weighing the importance of different channels. Another map in L_c_ uses a different set of weights to extract different channel combinations. The convolution operation is achieved by a single neuron scanning the input EEG data across the spatial domain with a local receptive field. The robustness of convolution operation to shifts and distortions of input is based on the property that if the input data shifted, the feature map output will be shifted accordingly.

After the convolution in the spatial domain, the spatial filters are detected. Then, the filtered data are convolved and subsampled in the temporal domain in L_cs_. The convolution operation is achieved by a single neuron scanning the input from the previous layer across the temporal domain with a 1 × K_m_ local receptive field. The subsampling operation can be achieved at the same time since the receptive fields of the contiguous neurons are non-overlapping. The convolution and subsampling combination tolerates the variance of the input to some degree because the reduction of spatial resolution can be compensated by the increase of the number of maps.

For layer L_c_ and layer L_cs_ in ConvEEGNN, each neuron in a layer receives inputs from a set of neurons located in a small neighborhood of the previous layer. By connecting neurons to local receptive fields on the previous map, neurons can extract elementary features like channel importance. The elementary feature detectors are useful on part of the previous map as well as across the entire map. Therefore, neurons in a map share the same set of weight vectors and perform the same operation even though the corresponding receptive fields are located at different places on the map. After a feature has been extracted, its approximate position relative to other features is more important to its exact location. To reduce the precision about the positions of features and obtain some degree of spatial or temporal invariance, convolutional layers are interspersed with subsampling layers. And layer L_cs_ combine the two operations in one layer.

### Input Normalization

The input I_i_ of ConvEEGNN is a matrix (similar to an image in ConvNet [29]), where each row of the matrix is a numeric time series of voltage measures for a channel. The size of I_i_ is N_ch_ × T, where T corresponds to the temporal sampling length. In our experiment, for the entire period, T is set to be 75 (25Hz × 3s, representing -0.1-2.9s). For pre-stimulus period and during-stimulus period, T_pre_ and T_dur_ are both set to be 30, representing 0.3-1.5s with 25Hz and 1.5-2.7s with 25Hz respectively.

First, the data are subsampled to the sampling frequency of 25 Hz in order to reduce the size of input. Many studies showed that memory performance is related to oscillatory activity in the theta (4-8 Hz) frequency band [13, 37–39]. Therefore, the subsampling operation provides most of the information relevant to memory performance. Then, the data are normalized with mean 0 and variance 1 to improve convergence during the learning of ConvEEGNN [40].

In this experiment, in total 30 channels are used. We exclude the horizontal electrooculogram and vertical electrooculogram since the two channels provide information to measure eye movement and are irrelevant to brain activity.

### Learning in ConvEEGNN

After the network topology of ConvEEGNN has been structured, we need to learn the weights of the network from training data. A typical process of learning consists of two main steps: feedforward and back-propagation [41]. For feedforward pass, the network processes the inputs according to the initial weights and provides resulting outputs. For back-propagation pass, the errors between the resulting outputs and the desired outputs corresponding to the input data are used to update the weights in order to gradually reduce the errors.

In this study, we extended the derivation and implementation of feedforward pass to ConvEEGNN. Let $k_{m}^{l}$ denote the kernel for the map m in layer l and $b_{m}^{l}$ denote the bias for the map m in layer l. Define output of the map m in layer L_c_ and L_cs_ to be:

$$\begin{array}{c} x_{m}^{L_{c}}=f\left(u_{m}^{L_{c}}\right),with\;u_{m}^{L_{c}}=conv\left(I_{i},k_{m}^{L_{c}},1\right)+b_{m}^{L_{c}}\;\;\;\;\;\; \end{array}$$ (3)

$$\begin{array}{c} x_{m}^{L_{cs}}=f\left(u_{m}^{L_{cs}}\right),with\;u_{m}^{L_{cs}}=conv\left(x_{m}^{L_{c}},k_{m}^{L_{cs}},K_{m}\right)+b_{m}^{L_{cs}} \end{array}$$ (4)

where *conv* is the convolution operation. For L_c_, input I_i_ is convolved with the kernel of $k_{m}^{L_{c}}$ and the convolution stride of one. For L_cs_, the output from layer L_c_ is convolved with the kernel of $k_{m}^{L_{cs}}$ and the convolution stride of K_m_ (the size of $k_{m}^{L_{cs}}$). Notice that a kernel is shared by each neuron of one map and layer L_cs_ has S/K_m_ neurons for each map. Then, the data are put through the activation function f(⋅) to form the output feature map. In L_c_, a kernel allows filtering in the spatial domain. In L_cs_, a kernel represents temporal filters and down-sampling, and this size of the data to analyze is reduced in this layer by performing convolution and subsampling at the same time.

The output of layer L_h_ and L_out_ can be achieved according to the typical feedforward pass of fully connected neural network [41]. Two neurons of the output layer, Z_0_ and Z_1_, represent the two classes. The input is predicted to be a forgotten event if the output of Z_0_ is larger than that of Z_1_, otherwise the input is recognized as a remembered one.

For each layer, the weights/kernels are initialized with a standard distribution around $\pm 1/n_{m}^{l}{\left(j\right)}_{input}$, where $n_{m}^{l}{\left(j\right)}_{input}$ is the number of inputs of the neuron j in the map m of layer l. The activation function f(⋅) for L_c_ and L_cs_ is hyperbolic tangent function. The constants are set with a = 1.7159 and b = 2/3, according to the recommendations described in [40]. The activation function for the last two layers is the logistic (sigmoid) function.

For back-propagation pass, we applied backpropagation algorithm [41] by minimizing the least mean square error. Like typical backpropagation pass, the resulting outputs are compared against the desired outputs corresponding to the input. And then, the errors are propagated back through the network to adjust the weights while the network is gradually converging on the ability to provide the desired outputs.

We use cross-validation for training and testing [42]. This method divides data into training data and testing data. For each division, the testing set is composed of one sample from each class (remembered or forgotten) and the remaining samples are used for training. The cross-validation process was repeated k times until each sample was used exactly once for testing. The k results from the k divisions can then be averaged to produce a single prediction accuracy. For the training procedure, the training samples are divided into a training set and a validation set, accounting for 70% and 30% respectively. To balance the number of samples for each class in the training set, we copied the samples of the smaller class [43]. The training stopped when the least mean square error was minimized on the validation set.

The average training time was around 4 minutes on a computer with an Intel Core i5-3470 CPU (3.20GHz) and 4GB RAM. The time depends on the number of training samples. The model was implemented in MATLAB without any special hardware optimization (multicore or GPU). The source codes will be available publicly if this paper is accepted. The average testing time was around 1 sec on the same computer.

## Results

In this section, we carried out four experiments to evaluate the performance of ConvEEGNN:

1. Test different network structures to find the optimal ConvEEGNN;
2. Compare ConvEEGNN with other approaches;
3. Evaluate the prediction results separately with pre-stimulus period and during-stimulus period to find out the contribution of different periods to memory performance;
4. Analyze contributions of different EEG channels with ConvEEGNN for prediction.

### Prediction Accuracy

We experimented with different network structures of ConvEEGNN. The structure is determined by the number of maps in the convolutional layer (N_c_) and the size of map m in the convolutional and subsampling layer (P_m_, m = 1, 2, … N_cs_). Table 1 shows the prediction results of different ConvEEGNN network structures for all 9 participants. In addition to the average prediction accuracy, significance based on total samples of each participant is also included. Significance means the number of significantly over chance results (significantly over 50% with p < 0.05) in all 9 participants [44, 45]. Higher significance suggests that an approach has higher accuracy. The average prediction accuracy varies nearly from 65% to 72%. The best accuracy of 72.07% was achieved with the network structure of N_c_ = 1, P_1_ = 3. For this network structure (N_c_ = 1, P_1_ = 3), all of the 9 participants showed prediction accuracies significantly over chance. The ConvEEGNN with different P_m_ in one network structure (N_c_ = 2, P_1_ = 3 & P_2_ = 5) achieved the accuracy of 70.15%, which is approximate to the best accuracy (72.07%) with high significance. The reason behind this may be that P_m_ is directly related to the size of kernel for the convolutional and subsampling layer. For this layer, the kernel convolved data in the temporal domain to extract sequential temporal features. The kernel size affects the range of time used for higher feature extraction. In view of the neural activity during memory process, kernel size may indicate the complexity for neurons related to memory formation to process EEG signals. Kernels with smaller size may indicate a relative simple signal process to extract short-time features about memory formation. Kernels with bigger size may indicate a relative complex signal process to extract long-time features about memory formation. The network structure of N_c_ = 2, P_1_ = 3 and P_2_ = 5 may take the advantage of combining these two kinds of features or signal processes and resulted in a high prediction accuracy (cf. Table 1).

**Table 1:** Prediction performance of different ConvEEGNN network structures.

Network Structure		Average Accuracy(%)	Significance
N_c_ = 1	P_1_ = 3	**72.07**	**9/9**
	P_1_ = 5	68.52	9/9
	P_1_ = 15	65.68	6/9
	P_1_ = 25	69.86	9/9
N_c_ = 2	P_1_ = 3 & P_2_ = 3	68.48	7/9
	P_1_ = 5 & P_2_ = 5	69.57	9/9
	P_1_ = 15 & P_2_ = 15	58.06	3/9
	P_1_ = 25 & P_2_ = 25	67.50	7/9
	P_1_ = 3 & P_2_ = 5	70.15	9/9
	P_1_ = 3 & P_2_ = 15	67.44	8/9
	P_1_ = 3 & P_2_ = 25	67.09	8/9

The significance column gives the proportion of the number of participants with significantly over chance results in all 9 participants.

### Comparison with Other Approaches

For comparison purposes, six other approaches were implemented and optimized, then tested on the same data with the same experimental protocol.

1. LDA: linear discriminant analysis.
2. ANN-1: one-hidden layer fully-connected artificial neural network. For ANN-1, the hidden layer has 10 neurons. Hyperbolic tangent function is used as the activation function of the first hidden layer and logistic (sigmoid) function is used for the other layer.
3. ANN-2: two-hidden layer fully-connected artificial neural network. For ANN-2, the two hidden layers have 20 and 10 neurons respectively. Hyperbolic tangent function is used as the activation function of the first hidden layer and logistic (sigmoid) function is used for the other layers.
4. SVM: support vector machine. After testing different kernels (linear, polynomial and radial basis function), we optimized the approach by using cubic polynomial as the kernel function.
5. SVM + LDA [20]: this classifier-fusion approach combined the results from two SVM classifiers for the spectral features of pre- and during-stimulus SME and an LDA classifier for the temporal features of during-stimulus SME. The kernel function for SVM is cubic polynomial.
6. CWT + SVM [21]: this approach used continuous wavelet transform to extract time-frequency features and then used 1-norm SVM to predict memory performance. Since the data were band-pass filtered between 0.05 and 15 Hz according to the structure of ConvEEGNN, the frequency bands extracted for 1-norm SVM are Theta 1 (centered at 4.00 Hz), Theta 2 (centered at 6.42 Hz) and Alpha (centered at 11.26 Hz).

As it can be seen in Table 2, ConvEEGNN outperformed all the other six approaches, suggesting that convolutional neural network may have some advantages over EEG analysis. By convolving across spatial and temporal domain, ConvEEGNN may be more robust to shifts or distortions of EEG signals. By subsampling in the temporal domain, the relative positions of features may be extracted to obtain some degree of temporal invariance [28, 46]. Since each neuron in a layer receives inputs from a set of neurons located in a small neighborhood of the previous layer, neurons may extract local fine grained features which benefits the signal analysis [46]. LDA was the worst model with the lowest accuracy and significance. Since LDA is good at classifying features with linear separability, the performance of LDA in Table 2 may suggest that the data have less linear separability. Among all the other approaches, SVM achieved relatively higher accuracy but the significance was similar to the other approaches, which was lower than the half number of the participants. From Table 2, SVM + LDA [20] showed average accuracy around 60% with low significance, which was outperformed by ConvEEGNN. This may suggest that the features exploited by ConvEEGNN might be more informative than those extracted by SVM + LDA [20].

**Table 2:** Prediction performance of various approaches.

Approach	Average Accuracy(%)	Significance
LDA	52.62	2/9
ANN-1	58.90	3/9
ANN-2	59.53	3/9
SVM	60.72	3/9
SVM + LDA [20]	60.97	4/9
CWT + SVM [21]	60.82	3/9
ConvEEGNN	72.07	9/9

The significance column gives the proportion of the number of participants with significantly over chance results in all 9 participants.

**Fig 4:**
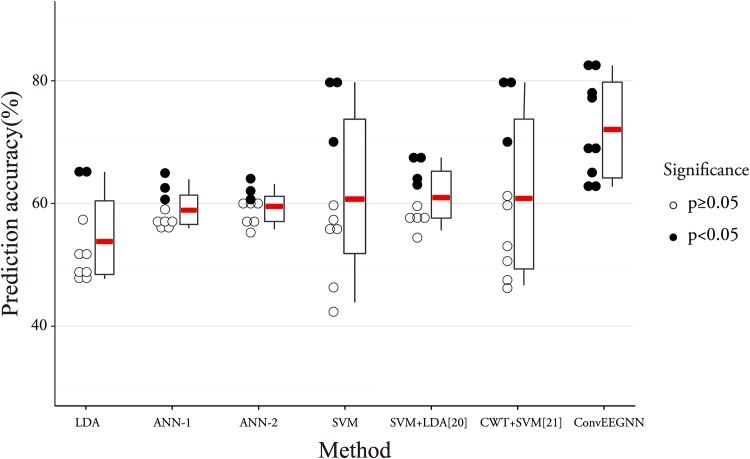
Detailed performances for all 9 participants using different approaches. The prediction accuracy and significance for ConvEEGNN are compared to: (1) LDA (2) ANN-1 (3) ANN-2 (4) SVM (5) SVM + LDA [20] (6) CWT + SVM [21]. The red bold line represents average prediction accuracy for each approach. The dots indicate the accuracies for every participant predicted by the approach next to it. Solid dot means that the prediction accuracy is significantly over chance otherwise soft dot is used.

**Fig 5:**
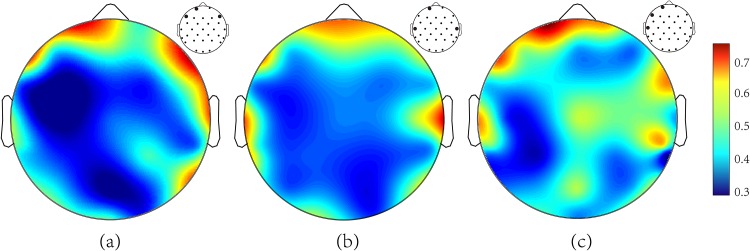
Weight map averaged across all 9 participants. (a) pre-stimulus period, (b) during-stimulus period, (c) entire period. The map is range-scaled. The contour maps show the position of all the channels used. The bold dots represent the top 3 channels for corresponding period.

Fig 4 detailedly shows prediction performance for all the participants using different approaches. For each approach, the upper and lower 25% quantiles of the accuracies for all 9 participants are represented with the box upper and lower boundaries, indicating the variance of accuracy for each approach. And high variance of accuracy means low stability of an approach. In Fig 5, compared to SVM, ConvEEGNN increased the average prediction accuracy without sharp increase in the variance of accuracy, which is similar to the variance of accuracy for SVM. This suggests that ConvEEGNN is relatively stable and accurate. The variance of accuracy for ANN-1 or ANN-2 was relatively low among all the approaches, revealing that the performance was stable, while the significance was low.

### Prediction Performance of Pre- and During-stimulus Periods

Since both EEG signals before an event [14, 15] and that during an event [11] have been found to reveal clues to distinguish remembered events from forgotten ones, in this subsection, we hope to investigate which kind of EEG signals contributes more for memory performance prediction. For that, EEG signals from the pre-stimulus period and during-stimulus period are taken separately as an input for the ConvEEGNN. In our experiment, the parameters standing for temporal sampling length for the pre-stimulus period and during-stimulus period, T_pre_ and T_dur_, were both set to 30, so as to be adapted to the structure of ConvEEGNN, representing 0.3-1.5s and 1.5-2.7s respectively. Therefore, the two inputs of the ConvEEGNN for the two periods were of the same size. The number of maps in the second layer and the size of maps in the third layer were in accordance with the network structure with the best performance mentioned before, which was N_c_ = 1, P_1_ = 3.

The performances for the two periods are compared with the performance for the entire period (-0.1-2.9s) in Table 3, which shows the prediction accuracy as well as significance for all 9 participants. From Table 3, we can see that the prediction performance with pre-stimulus signals and that with during-stimulus signals is very close and both of them are nearly 67%, significantly over chance for at least eight participants. This result may indicate that pre-stimulus period and during-stimulus period have very similar contribution for subsequent memory performance prediction. The information from pre-stimulus period and during-stimulus period may have similar relation with memory process. Compared to average accuracy using single period of EEG data, the average accuracy using the entire period increases approximately 5%, which is significantly better than either the pre-stimulus period (t-score < 0.01) or the during-stimulus period (t-score < 0.05). And each participant’s accuracy is significantly over chance for the entire period.

**Table 3:** Prediction accuracy using pre-stimulus, during-stimulus and entire period.

Participant	Pre-stimulus Period	During-stimulus Period	Entire Period
P01	61.62*	61.62*	62.63*
P02	68.42*	77.19*	77.19*
P11	62.00*	63.00*	65.00*
P12	68.00*	71.00*	78.00*
P13	80.00*	81.25*	82.50*
P14	82.47*	70.10*	82.47*
P16	60.67*	59.55	62.92*
P20	62.69*	64.18*	68.66*
P22	65.38*	61.54*	69.23*
Average	67.92	67.71	72.07

The superscript * means significantly over chance result (significantly over 50% with p < 0.05).

### EEG Channel Analysis with ConvEEGNN

To infer the influence of different channels for prediction, the weights from the input layer to the second layer of the ConvEEGNN were examined. The absolute value of a weight provides a channel’s discriminant capability for telling remembered events from forgotten ones. Higher absolute value means higher discriminant capability. Fig 5 shows the weight maps of EEG channels averaged over all the participants for pre-stimulus, during-stimulus and entire period respectively. The red means a high absolute value while the blue represents a low weight. Table 4 shows the top three channels for all the three periods.

**Table 4:** Top-3 channels for pre-stimulus period, during-stimulus period and entire period.

Period	Pre-stimulus Period	During-stimulus Period	Entire Period
Top-3 channels	Fp1 F7 F8	Fp1 T7 T8	Fp1 F7 T7

For the pre-stimulus period, the largest weight was from the channels over prefrontal cortex. In other words, the discriminant capability over prefrontal cortex was the highest. For the during-stimulus and entire period, the greatest weights were from the signal sources above the prefrontal and temporal cortex (Fp1 and T7) (cf. Fig 5b and 5c). The discriminant channels are inferred by the weights in the data-driven ConvEEGNN. We find that these results are in accordance with the distribution of SME, that is, the magnitude over prefrontal cortex is the largest [14, 15]. This indicates the rationality of our approach somewhat.

## Conclusion & Discussion

In this paper, we proposed a computational approach called ConvEEGNN to predict memory performance using EEG signals. The ConvEEGNN can automatically extract features and integrate them with classification. The effectiveness of the proposed approach was validated by the recorded EEG signals in an auditory memory task. The results demonstrated that ConvEEGNN is effective to estimate earlier than his/her actual memory performance, outperforming other typical approaches. It was also found that EEG signals from pre-stimulus period and those from during-stimulus period have the very similar prediction accuracy.

ConvEEGNN has some underlying advantages for EEG-based memory prediction. ConvEEGNN allows automatic feature extraction via end-to-end training within the convolutional layers and the subsampling layers. This is helpful for EEG signal analysis since the signal contains many variations over time. By convolving, ConvEEGNN can be more robust to shifts or distortions of the input data. By subsampling in the temporal domain, the relative positions of features can be extracted to obtain some degree of temporal invariance [28, 46]. In addition, since each neuron in a layer receives inputs from a set of neurons located in a small neighborhood of the previous layer, neurons may extract local fine grained features or some kinds of underlying features which benefits the signal analysis [46]. For example, in ConvEEGNN, the kernel size, that is the number of neurons in the network used for convolving or subsampling, may indicate the complexity for neurons about memory formation to process EEG signals. In this way, kernels with smaller size may indicate a relative simple signal process to extract short-time features about memory. Kernels with bigger size may indicate a relative complex signal process to extract long-time features. By combining these two kinds of features, the network structure with different kernel size could achieve a relatively high prediction accuracy.

Compared to standard convolutional neural network for image recognition [29], kernels used in ConvEEGNN are vectors but not matrices, in order to separately extract spatial features crossing channels and temporal features in a single channel. We notice that the input of ConvEEGNN is a matrix which includes both spatial and temporal dimension. A vector kernel can convolve over only one dimension (i.e. spatial or temporal), thus only one kind of features (spatial or temporal) could be extracted. However, if a matrix kernel is used, it will convolve over not only spatial dimension but also temporal dimension, which will result in the features combining spatial and temporal domain. Separation of spatial and temporal domain has a distinctive advantage that it is easy to explain and understandable to optimize. For example, with spatial features, we can easily find which channel is more significant for remembering performance, and which is less.

This study still is limited by its participant number. Since only 9 participants were used for prediction, the data for training an optimal structure of ConvEEGNN were limited in number. Therefore, the ConvEEGNN that we optimized in this study is relatively limited in its prediction performance. As a matter of fact, CNN has advantages in modeling in various fields, such as speech recognition [36] and image classification [29]. Its advantages would be strengthened given more data. If more participants were provided, we might take full advantages of CNN to achieve a better prediction performance with a more complex structure of ConvEEGNN.

The weights from the input layer to the second layer of ConvEEGNN showed the influence of different channels for prediction. The results are consistent with the distribution of SME [14, 15]. In addition, the results for the during-stimulus and entire period may reveal a potential relation between the channels over temporal cortex and memory process.

Memory performance prediction has various applications. For instance, it could help us remember new knowledge better, and then improve learning efficiency. It may help diagnosis and treatment of those diseases regarding memory symptom, such as mild cognitive impairment and Alzheimer’s disease. It may also be very helpful to build a brain-in-loop system for cyborg intelligence [47–49].
